# Nest usurpation: a specialised hunting strategy used to overcome dangerous spider prey

**DOI:** 10.1038/s41598-019-41664-6

**Published:** 2019-03-29

**Authors:** Ondřej Michálek, Yael Lubin, Stano Pekár

**Affiliations:** 1 Department of Botany and Zoology, Faculty of Science, Masaryk University, Kotlářská 2, 611 37, Brno, Czech Republic; 2 The Blaustein Institutes for Desert Research, Ben-Gurion University of the Negev, Midreshet Ben‐Gurion, Israel

## Abstract

Hunting other predators is dangerous, as the tables can turn and the hunter may become the hunted. Specialized araneophagic (spider eating) predators have evolved intriguing hunting strategies that allow them to invade spiders’ webs by adopting a stealthy approach or using aggressive mimicry. Here, we present a newly discovered, specialized hunting strategy of the araneophagic spider *Poecilochroa senilis* (Araneae: Gnaphosidae), which forces its way into the silk retreat of the potential spider prey and immobilizes it by swathing gluey silk onto its forelegs and mouthparts. *Poecilochroa senilis* has been reported from the nests of a several, often large, spider species in the Negev desert (Israel), suggesting specialization on spiders as prey. Nevertheless, in laboratory experiments, we found that *P*. *senilis* has a wider trophic niche, and fed readily on several small insect species. The specialized nest-invading attack was used more frequently with large spiders, and even small juvenile *P*. *senilis* were able to attack and subdue larger spiders. Our observations show that specific hunting tactics, like nest usurpation, allow specialized predators to overcome defences of dangerous prey.

## Introduction

Evolutionary arms races between prey and predators lead to the evolution of various defence mechanisms of the prey and counter-adaptations of predators to subdue such a prey^1^. Predator-prey arms races are often asymmetrical, as a prey organism is under stronger selection pressure^2^. As a result, prey possess various primary and secondary defences^3^ that make them hard to capture or even dangerous for a predator. Hunting of such prey may be too costly for a predator, as it faces a risk of injury or even death – for example, a porcupine can badly injure its predators with quills, or stingrays may even kill potential predators with a poisoned spine^4^. The danger imposed by a prey may balance the selection pressures acting on prey and predator, resulting in avoidance of such prey, or the evolution of effective counter-adaptations of a predator. For example, one garter snake species that feeds on amphibians has evolved resistance to the toxins of a poisonous newt^2^.

Spiders are the most numerous terrestrial predators^5^, possessing venom and silk, which makes them dangerous; but they are also prey of many other predators. Many spiders use silk to construct nests, retreats, and barrier webs that may serve as primary protective devices against their predators, including other spiders^6^. Shelter construction was shown to protect spiders from lizard predation^7^, but shelters are not universal barriers against all predators. Firstly, various defence mechanisms may be perceived differently by arthropod and vertebrate predators^6^. Secondly, specialized arthropod predators have evolved behavioural adaptations allowing them to penetrate these barriers and to trick dangerous resident prey. For example, predators specialized to hunt web-building spiders may use aggressive mimicry to deceive their victims. When entering a web of their prey, they mimic the vibrations of caught prey or of a potential mate^8–11^. Other specialized predators are able to invade alien webs using different tactics, such as stealthy approach^12,13^.

**Figure 1:**
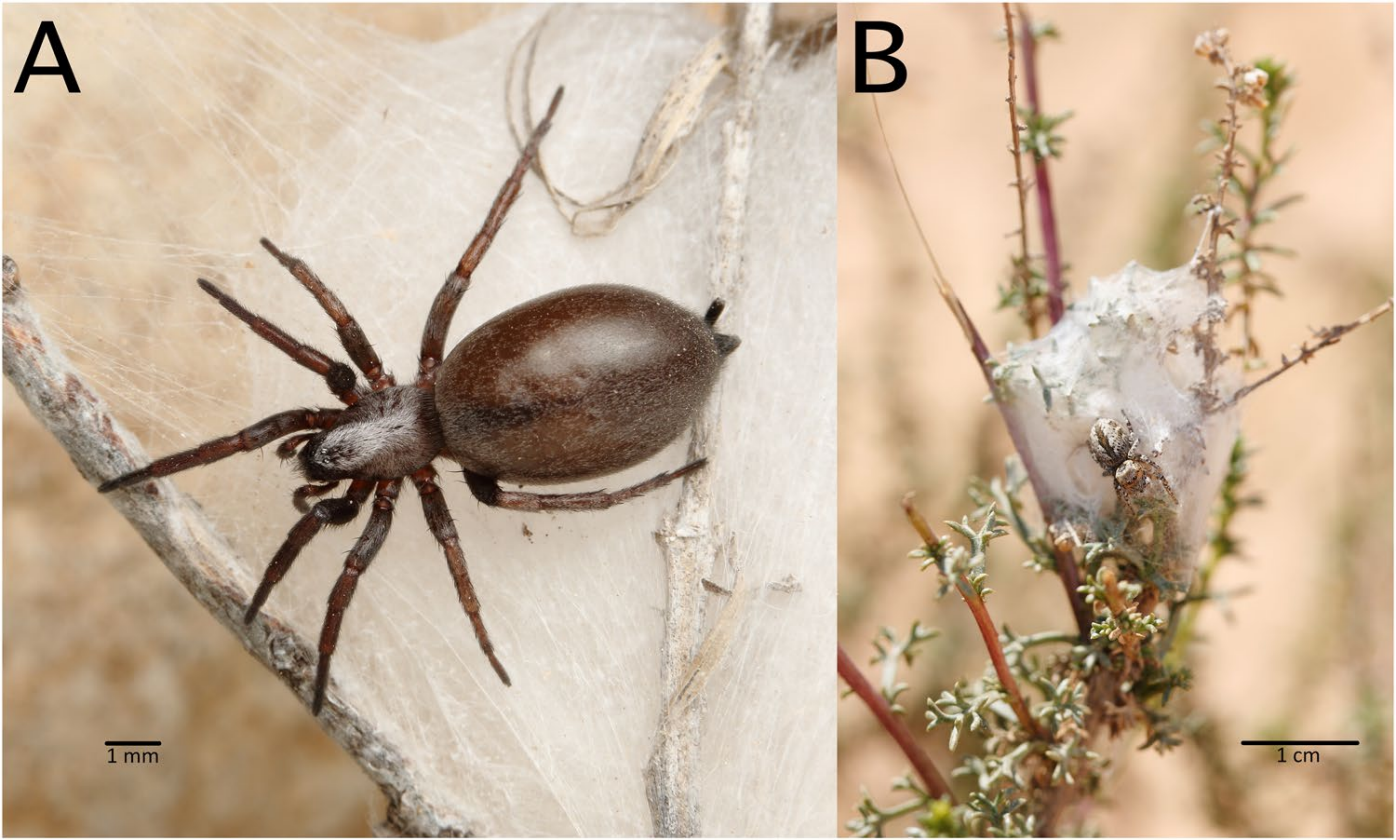
Studied spiders. (**A**) A *Poecilochroa senilis* female on the nest of *Mogrus logunovi*. (**B**) The nest of a *Mogrus logunovi* female with the resident spider sitting on the nest. Photos: O. Michálek.

Notably, araneophagy (predation on spiders) and web invasion have been observed in several species in the Gnaphosidae^14^, a family of largely nocturnal, active hunting spiders that do not build a capture web. In the Negev desert, Israel, the gnaphosid spider *Poecilochroa senilis* (O. Pickard-Cambridge, 1872) (Fig. 1A, further shortened to *Poecilochroa*) was found frequently in the retreats of other spiders, especially of web-building species such as the widow spider *Latrodectus revivensis* Shulov, 1948 (Theridiidae) and the velvet spider *Stegodyphus lineatus* (Latreille, 1817) (Eresidae), and in the retreats of cursorial jumping spiders, *Mogrus* spp. (Salticidae)^15^. However, it is unclear whether *P*. *senilis* is a predator of these spiders, or whether its presence in their retreats is an act of the secondary use of shelters. Previous research on other closely-related species^8^ suggests that *P*. *senilis* might be a predator that ambushes other spiders in their retreats.

The aim of our study was to investigate the trophic ecology of *Poecilochroa* to determine the nature of the interaction between *Poecilochroa* and its hosts. We tested the hypothesis that *Poecilochroa* is a predator of shelter-building spiders. Alternatively, but not mutually exclusively, *Poecilochroa* might utilize empty shelters as retreats. First, we investigated whether its fundamental trophic niche includes spiders. We then conducted observations to reveal whether it uses aggressive mimicry or another deception strategy to usurp the nest and the host, represented by the jumping spider *Mogrus logunovi* Prószynski, 2000 (Fig. 1B, further shortened to *Mogrus*). Given the fact that the host species are larger than *Poecilochroa*, we anticipated the use of a specialised capture strategy.

## Results

### Fundamental trophic niche

**Figure 2:**
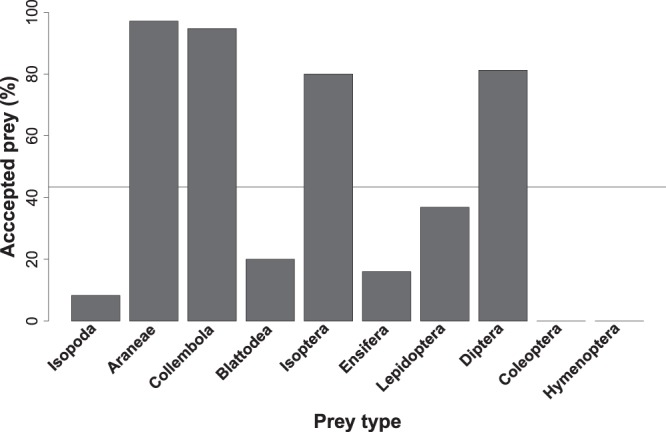
Comparison of the relative frequencies (in percentages) with which ten prey types were accepted by *Poecilochroa senilis* in the laboratory. The horizontal line shows the overall mean of prey acceptance.

*Poecilochroa* accepted eight out of the ten prey orders offered, but at significantly different frequencies (GEE-b, χ^2^_9_ = 27594, P < 0.0001, Fig. 2). *Poecilochroa* did not accept beetles or ants. Three prey types were accepted at a significantly lower frequency than average: woodlice, cockroaches, and crickets (Binomial tests, P < 0.04). Caterpillars were accepted at the average frequency (Binomial test, P = 0.7). Four prey types were accepted at a significantly higher frequency than average: spiders, springtails, termites and fruit flies (Binomial tests, P < 0.001). These results indicate that *Poecilochroa* is araneophagous, but not exclusively so. Levins’ index of niche breadth indicated an intermediate niche breadth (B_A_ = 0.52).

### Capture efficiency

**Figure 3:**
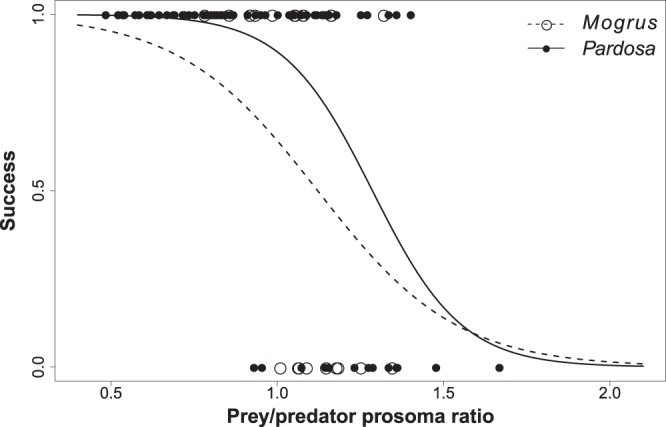
Comparison of the capture success of *Poecilochroa senilis* on two spider prey, *Mogrus* inside the nest and *Pardosa* outside the nest, related to the various relative prey/predator sizes. Estimated logit models are shown.

The capture success of prey spiders varied significantly with the relative prey/predator size ratio (GEE-b, χ^2^_1_ = 34.2, P < 0.001). *Poecilochroa* individuals were still able to successfully capture offered wolf spiders (*Pardosa* sp.) in the half of the cases when a relative prey/predator (prosoma) size ratio was equal to 1.29 (i.e. wolf spiders were larger than *Poecilochroa*) (Fig. 3).

The first instar juveniles of *Poecilochroa* subdued *Mogrus* juveniles in 93% of the trials (N = 45), despite the fact that *Mogrus* juveniles were always larger than *Poecilochroa* (mean *Mogrus*/*Poecilochroa* size ratio: 1.25 ± 0.11).

### Nest usurpation

**Figure 4:**
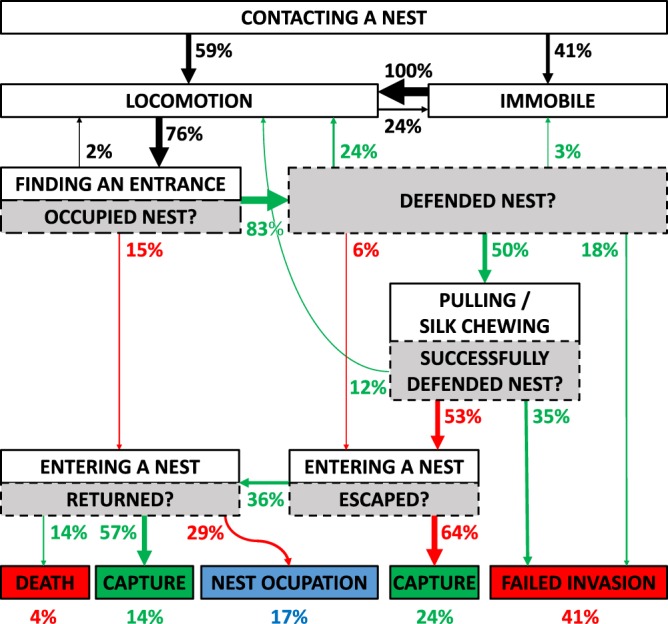
Flow diagram of the behaviour of *Poecilochroa senilis* on *Mogrus logunovi* nests. White boxes represent behaviours of *Poecilochroa*; grey boxes represent responses of *Mogrus*. Successive green arrows represent positive responses of *Mogrus* (“yes”) and red arrows represent negative responses of *Mogrus* (“no”), followed by subsequent *Poecilochroa* behaviour. Transition probabilities are represented by the line width of arrows and percentages. Coloured boxes represent the outcomes of hunting sequences: green – a successful hunt; red – a failed hunt; blue - unresolved. There were four possible outcomes of the interaction between *Poecilochroa* and *Mogrus*; capture - *Poecilochroa* captured *Mogrus*; death – *Poecilochroa* was killed by *Mogrus*; nest occupation - *Poecilochroa* remained inside the empty nest; failed invasion – *Poecilochroa* retreated from the nest or stayed immobile on the nest after its unsuccessful invasion, while *Mogrus* remained inside the nest. Numbers of percent below the diagram represent the proportion of sequences resulting in a given outcome. The flow diagram was made using the ethogram (Table 1) and a transition matrix based on the ‘Nest usurpation’ experiment.

*Poecilochroa* used a repertoire of behaviours to usurp a *Mogrus* nest (Table 1). Once *Poecilochroa* contacted the silk of a *Mogrus* nest, it either continued to move on the nest or paused. Sometimes *Mogrus* abandoned the nest while *Poecilochroa* was walking or standing on the nest. *Poecilochroa* occasionally plucked the nest silk, i.e. repeatedly pulling at it sharply with one or several legs, or shivered, i.e. bouncing its body with all legs in contact with the silk. *Mogrus* showed no visible response to these movements. *Poecilochroa* continued to move on the nest, accompanied by pauses, until it found one of the two nest entrances. If the nest was empty, *Poecilochroa* moved inside and remained there. When *Poecilochroa* tried to enter an occupied nest, *Mogrus* usually defended its nest by pulling in the silk at the entrance, and thus closing it, or by simply blocking the entrance. *Poecilochroa* tried to penetrate this defence by pulling or chewing the silk. If *Mogrus* failed to defend the nest, *Poecilochroa* crawled inside and *Mogrus* either was killed or escaped via the second entrance (Fig. 4, Video S1).

**Table 1:** Ethogram of behaviours observed in *Poecilochroa senilis* while invading the nest of *Mogrus logunovi* (**A**) and responses of *M*. *logunovi* during the invasion of *P*. *senilis* (**B**).

A. Behaviour of *Poecilochroa*	Description
Contacting a nest	Contacted the silk of the nest.
Locomotion	Walked on the nest or the branch.
Remaining immobile	Stopped without further locomotion.
Finding an entrance	Encountered one of the two nest entrances.
Pulling	Pulled the silk with legs I and/or II.
Silk chewing	Chewed the silk.
Entering a nest	Crawled inside the nest.
**B**. **Response of** ***Mogrus***	**Description**
Occupied nest?	Was it present inside the nest?
Defended nest?	Was it defending its nest?
Successfully defended nest?	Was it successful in repelling *Poecilochroa?*
Escaped?	Did it escape from its nest?
Returned?	Did it return to its nest after leaving?

*Poecilochroa* individuals attempted to invade occupied *Mogrus* nests in 91% of all observations (N = 32). In 9%, *Poecilochroa* made its own silken retreat within the box away from the *Mogrus* nest and did not attempt to enter the nest. *Poecilochroa* invaded the nest and captured *Mogrus* in 38% of all usurpation attempts (N = 29); either immediately after entering the nest (24%), or after *Mogrus* escaped the nest but returned within 24 hours (14%). In 17%, *Poecilochroa* also invaded the nest, but *Mogrus* escaped and did not return within 24 hours. In 41% of usurpation attempts, *Mogrus* was able to defend its nest successfully, and in one case (4%) *Mogrus* even killed *Poecilochroa* (Fig. 4).

Capture success on nests declined with the relative size ratio of the prey and the predator, as larger *Mogrus* individuals were better able to defend the nest. In addition, it differed from the capture success with wolf spiders as prey (GEE-b, χ^2^_1_ = 4.2, P = 0.04), as *Poecilochroa* invaded occupied nests with a 50% capture success rate at a lower relative body ratio equal to 1.12 (Fig. 3). Thus *Poecilochroa* was less effective in capturing *Mogrus* than in capturing wolf spiders that were captured with a same success rate at a higher body ratio equal to 1.29.

When presented with an empty *Mogrus* nest, 79% of *Poecilochroa* individuals (N = 19) entered the nest and remained inside after the first hour, with even more individuals (95%) occupying the nest after 24 hours.

### Predatory behaviour

**Figure 5:**
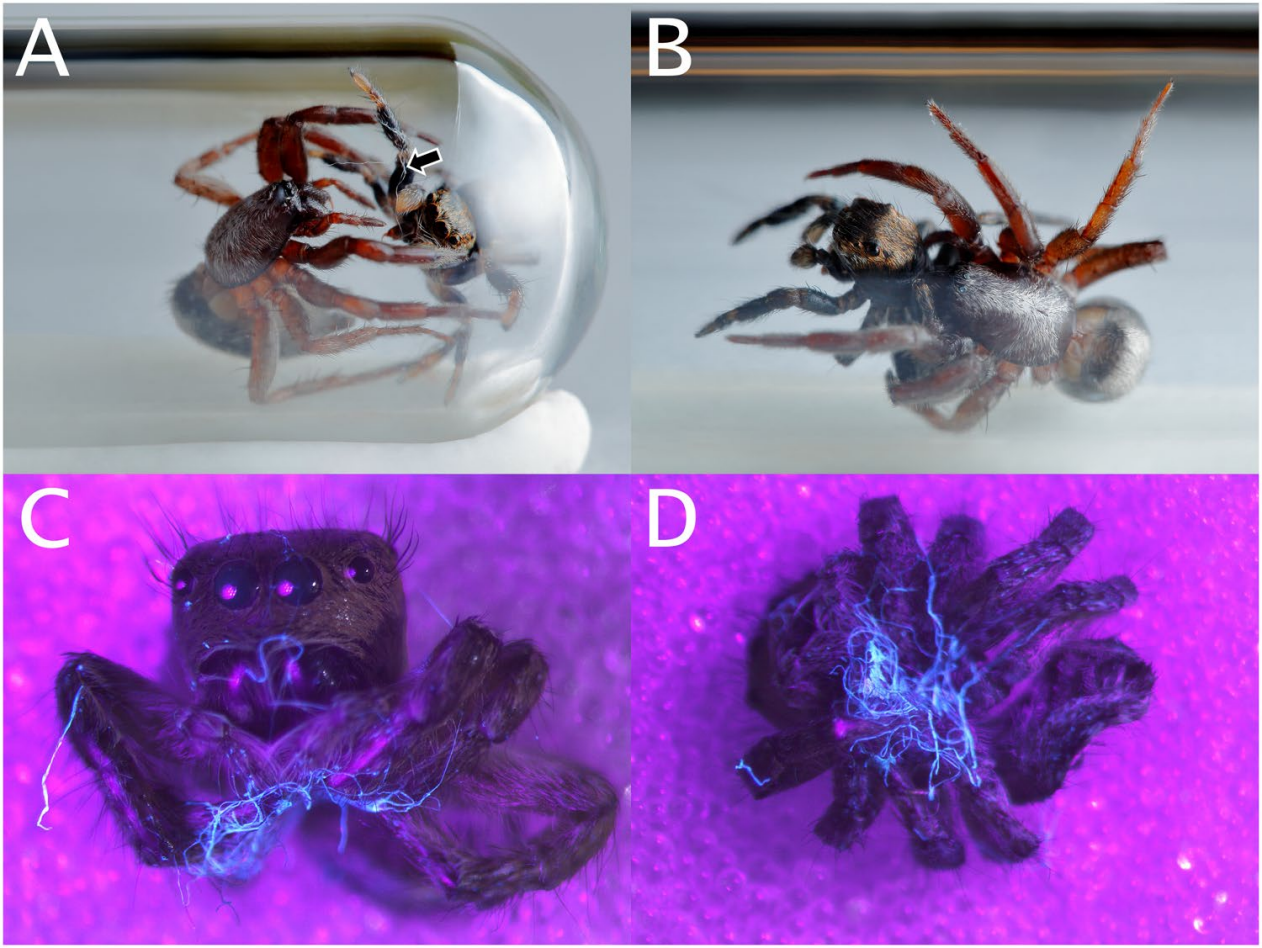
Silk swathing during prey capture by *Poecilochroa senilis*. (**A**) *Poecilochroa* (on the left) facing the jumping spider (on the right) with curled opisthosoma towards the jumping spider and swathing piriform silk on its forelegs and mouthparts. Note the piriform silk strand attached to the jumping spider’s right foreleg and palp (arrow). (**B**) The immobilized jumping spider is afterwards paralyzed by a bite to the anterior part of the opisthosoma. (**C**,**D**) Frontal (**C**) and ventral (**D**) view of the jumping spider *M*. *logunovi* after being captured by *P*. *senilis*. Piriform silk is white/blue under UV light. Photos: O. Michálek.

**Figure 6:**
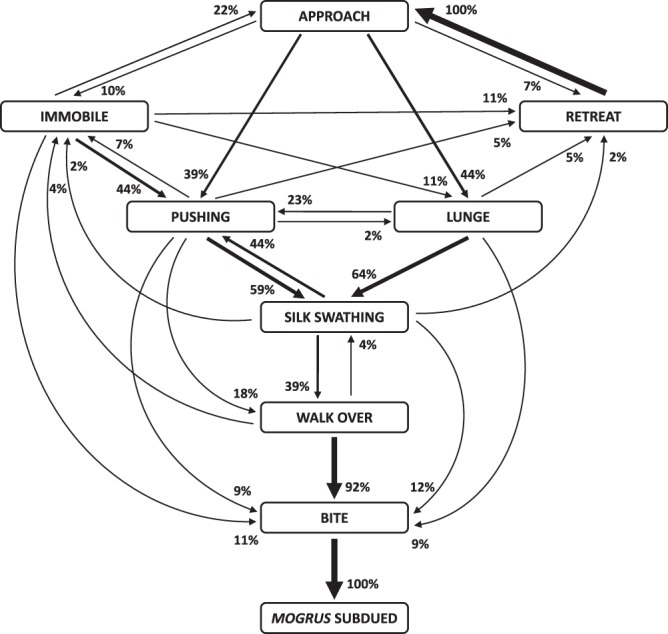
Flow diagram of the prey capture behaviour of *Poecilochroa senilis* using *Mogrus logunovi* outside its nest as prey. Transition probabilities are represented by the line width of arrows and percentages. The flow diagram was made using the ethogram (Table 2) and a transition matrix based on the ‘Predatory behaviour’ experiment.

**Figure 7:**
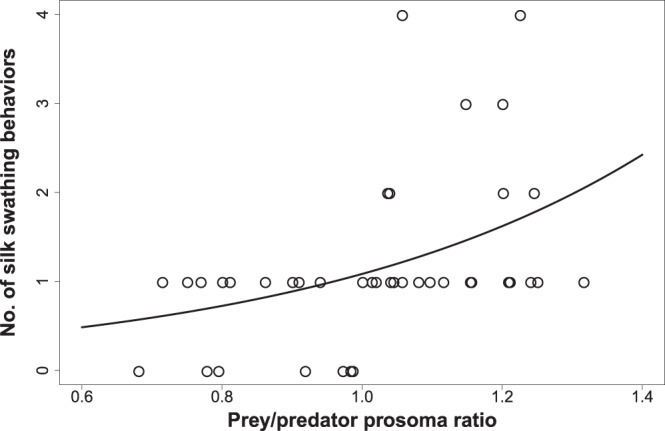
Relationship between the number of silk swathing events used by *Poecilochroa senilis* while hunting *Mogrus logunovi* jumping spiders and various relative prey/predator sizes. Estimated model is shown.

*Poecilochroa* used a range of behaviours to subdue *Mogrus* after a direct contact (Table 2). When approaching *Mogrus*, *Poecilochroa* usually lunged at it or pushed it with its forelegs so that *Mogrus* could not lunge back at *Poecilochroa*. If *Mogrus* resisted, *Poecilochroa* curled its opisthosoma ventrally towards *Mogrus* and extruded gluey silk from its piriform glands onto the prey’s forelegs and mouthparts to immobilize it (Fig. 5). Hunting sequences typically ended with *Poecilochroa* walking over *Mogrus* and biting it (Fig. 6, Video S2). The number of silk swathing events in a single hunting sequence significantly increased with the relative size of the prey (GEE-p, χ^2^_1_ = 11, P < 0.001, Fig. 7). *Poecilochroa* occasionally hunted relatively smaller *Mogrus* spiders without the use of piriform silk, while in several observations it applied the silk repeatedly on larger *Mogrus* spiders (Fig. 5). A similar silk swathing attack was also used on fleeing or resisting wolf spiders.

**Table 2:** Ethogram of prey-capture behaviours observed in *Poecilochroa senilis* while overcoming *Mogrus logunovi* spiders.

Behaviour	Description
Approach	*Poecilochroa* or *Mogrus* moved toward the other spider.
Immobile	*Poecilochroa* stopped and remained briefly immobile.
Retreat	*Poecilochroa* or *Mogrus* moved away from the other.
Lunge	*Poecilochroa* lunged towards *Mogrus* by rapidly extending legs III and IV.
Pushing	*Poecilochroa* pushed the prosoma of *Mogrus* with elevated legs I and II.
Silk swathing	*Poecilochroa* swathed the piriform silk on *Mogrus* forelegs and mouthparts.
Walk over	*Poecilochroa* walked over *Mogrus*.
Bite	*Poecilochroa* delivered a bite to *Mogrus* and held it until it was paralyzed.

## Discussion

We show here that the fundamental trophic niche of the spider *Poecilochroa senilis* is moderately wide. Spiders were one of the preferred prey types, although not the only one, suggesting that *Poecilochroa* is moderately stenophagous. Prey specialization among araneophages, however, is not usually strict, as they often accept several alternative prey types beside spiders, though at lower frequencies^16–20^. We investigated the trophic niche in juveniles of *P*. *senilis* only, because the number of adult individuals collected in the field was very low for the experiment. Although the trophic niche breadth may increase with age of spiders as bigger spiders can hunt bigger prey^21^, specialized spiders hunting dangerous prey like ants or other spiders are usually able to subdue larger prey than themselves even as juveniles^22–24^. Similarly, *Poecilochroa* was able to overcome larger spiders than itself as a juvenile. Its hunting strategy towards spider prey was therefore very effective, indicating prey-specialised hunting behaviour.

In nature, in addition to the nest-building salticid *M*. *logunovi*, *Poecilochroa* was found to attack and feed on two species of web-building spiders by capturing them in their nests, namely the widow spider *Latrodectus revivensis* and the cribellate spider *Stegodyphus lineatus*^15^. These observations support our laboratory experiments indicating that *Poecilochroa* is able to handle a rather broad range of large and dangerous spider prey. The extent to which it feeds on insects in nature is unknown. However, spiders are especially numerous arthropods in desert ecosystems, both in terms of abundance and biomass^25–27^. Intraguild predation (predation among predators) plays an important role in shaping the composition of desert arthropod communities^27,28^, as more than 50% of the diet of desert predatory arthropods is composed of other predators; and predatory arthropods constitute a high proportion of all desert arthropods^29^. We collected *Poecilochroa* solely in association with other spiders, suggesting that it is locally specialized on these abundant prey in the desert.

We found that *Poecilochroa* is able to subdue *Mogrus* inside the nest. Entering a spider web or nest is dangerous, as it is an extension of the spider’s senses and any intruder can become a prey. Web-invaders usually use several approaches to avoid this: they either deceive their victim by means of aggressive mimicry^8–11^, by approaching the prey stealthily^12,13^, or by leaping suddenly onto webs from a distance^30,31^. *Poecilochroa* used a very different approach – namely, rapid nest usurpation: once it found the entrance of the nest, it tried to enter and swiftly subdue the resident spider without using stealth. A similar hunting tactic was observed in the spider *Nyssus coloripes* Walckenaer, 1805 (Corinnidae), which relied on its rapid, erratic style of locomotion when invading webs^32^, and *Zelanda erebus* (L. Koch, 1873) (Gnaphosidae), which was able to chew through silk and force its way inside the nests of jumping spiders. However, *Z*. *erebus* also utilized aggressive mimicry when invading webs^8^. Although *Poecilochroa* sometimes moved its body and legs during locomotion or when standing on the nest in a way that may produce vibrations, *Mogrus* was not lured out of the nest in search of a prospective prey. However, as *Mogrus* sometimes left the nest before *Poecilochroa* invaded it – that is, after it had become aware of *Poecilochroa*’s presence (Michálek, pers. obs.) – such leg movements may have been a way of checking whether *Mogrus* was present in the nest. We cannot, however, rule out the possibility that *Poecilochroa* utilizes other tactics when invading the webs of other species. In this respect, we tried to observe invasions of the nests of adult *Stegodyphus lineatus*, but *Poecilochroa* did not attack these spiders in the web at all (Michálek, pers. obs.), possibly because the *S*. *lineatus* individuals were too large. Nevertheless, *Poecilochroa* may shift to attacking *S*. *lineatus* during the season when these spiders are juveniles^15^.

*Poecilochroa* was not always successful in invading nests. It was able to subdue large spiders when hunting outside nests, but was less successful in capturing them in their shelters. As a key element in overcoming large prey is immobilization with piriform silk, it seems that *Poecilochroa* is limited in the use of this silk when entering the narrow *Mogrus* nest. A conditional strategy for hunting larger *Mogrus* spiders may be to wait outside the nest and ambush *Mogrus* when it leaves the nest. However, the prey may more easily escape in the open space outside the nest. Alternatively, *Poecilochroa* may enter an empty *Mogrus* nest while *Mogrus* is foraging and ambush *Mogrus* when it returns, as we also observed.

When a resident *Mogrus* escaped and was not captured later, *Poecilochroa* often remained inside the host nest at least for one day. It is possible *Poecilochroa* utilize empty shelters even for longer periods, as we found *Poecilochroa* exclusively inside *Mogrus* nests during our field survey. Usurping webs or nests may have other benefits^8^; in this case, *Poecilochroa* may utilize the *Mogrus* nest as a shelter, feed on the eggs or young of the resident spider (Michálek, pers. obs.), or use the nest for its own oviposition^15^.

*Poecilochroa* utilized gluey piriform silk to immobilize spider prey. Web-building spiders use piriform silk as attachment discs^33^, but gnaphosid spiders possess a modified spinning apparatus allowing them to use piriform silk as adhesive tape and to hunt dangerous prey, such as other spiders^34^. We observed that *Poecilochroa* used silk conditionally depending on the relative prey size. *Poecilochroa* was able to subdue relatively smaller spiders without the use of swathing silk, while it applied silk repeatedly when immobilizing larger spiders. It thus showed a high level of versatility on prey type and size. Specialized spiders often display stereotyped hunting behaviour^23,24^. Several araneophagous spiders, however, utilize a variety of tactics depending on the predatory context^8,32,35^. As spider-eating spiders are usually not as strictly specialized as ant or termite specialists^36,37^, araneophagous spiders may retain greater plasticity in their hunting behaviour. Some specialized spiders have an innate search image of their focal prey, or the search image is formed in a single encounter^38,39^. *Poecilochroa* readily attacked and preyed upon *Mogrus* even as a naïve juvenile, when this prey type was novel to it, and it was already able to subdue large prey.

A flexible line of defences from multiple primary to secondary strategies has evolved in prey that allow them to avoid or deter predators^40^. Anti-predator strategies may be also influenced by the individual’s condition, for example, its reproductive state^41^. Therefore, a predator is confronted with a diversity of prey defensive mechanisms. A successful predator must have a versatile predatory behaviour that allows it to overcome all subsequent defences of a prey. This is even more pronounced when the prey is a potential predator as well. Here, we have shown *Poecilochroa* maintains such versatility by overcoming both primary and secondary defences of its dangerous spider prey: it penetrates the defended shelter by pulling and chewing silk, ambushes the spider fleeing from shelter, usurps and utilizes the empty shelter, and immobilizes dangerous, resisting prey with silk.

## Methods

### Studied species

During our survey in the Negev desert, we found nests of the jumping spider *M*. *logunovi* occupied by *Poecilochroa* (Michálek & Pekár, pers. obs.). Therefore, we focused on the interaction between these two species. *Poecilochroa* individuals (Fig. 1A) were collected at Mashabim (31°00′07.3″N, 34°45′18.3″E) and Retamim (31°06′27.5″N, 34°39′15.0″E) sand dunes in the Negev desert, Israel, in April 2016 and in March and April 2017. Individuals were collected by inspecting nests of *Mogrus* found on different shrub species (mainly *Retama retama*, *Artemisia monosperma*, and *Thymelaea hirsuta*). *Mogrus* individuals (Fig. 1B) were collected in 2017, along with their nests, on shrubs at the same localities as *Poecilochroa*. *Mogrus* nests are composed of several layers of silk, forming a ‘sleeping bag’ with two openings, one at each end (Fig. 1B). Only 1.4% of the 510 *Mogrus* nests examined during March and April 2017 were found to contain *Poecilochroa* spiders. Some females of *Poecilochroa* collected in the field produced egg-sacs in the lab. Hatched juveniles were also used in experiments along with individuals collected in the field.

*Poecilochroa* spiders were kept in plastic vials (length 55 mm, diameter 12 mm) containing moistened gypsum, and stored in a chamber at constant temperature (22 ± 1 °C) and under a 16:8 LD regime. *Mogrus* spiders were kept in plastic containers (55 × 55 × 75 mm) along with their nests at room temperature (22 °C) and under a natural 14:10 LD regime. All spiders were fed at least once a week with *Drosophila* flies *ad libitum* or were allowed to consume the prey accepted in laboratory trials to standardize their satiation level. Prey used in experiments were either laboratory reared or collected around the university campus in Brno, Czech Republic and kept at 10 °C before using in the experiment. Experiments were performed from August 2016 to April 2018 in Israel and in the Czech Republic. All statistical analyses were performed in the R environment^42^.

### Fundamental trophic niche

To investigate the fundamental trophic niche of *Poecilochroa*, prey acceptance experiments^43^ were performed with 39 juvenile *Poecilochroa* individuals. Spiders were starved for one week before being used in trials. Individuals were placed singly in Petri dishes (diameter 50 mm). The trials began after at least 1 h of acclimation. Ten prey types (Table 3) were offered to each spider in a randomised order. Each prey type was offered to each individual spider only once. If the prey was not attacked within one hour, it was replaced with a different prey type. The trial ended when a spider had killed and consumed a prey. If a spider did not accept any prey type, it was considered to be unmotivated to eat (e.g. satiated or preparing to moult) and data from such trials were rejected. Trials were performed at one-week intervals for each individual.

**Table 3:** List of prey types used in acceptance experiments, their body sizes (*body size, **prosoma size), and the number of *Poecilochroa* individuals to which was offered given prey (N).

Order/Family	Species	Prey size (mm)	N
Isopoda/Armadillidiidae	*Armadillidium vulgare* Latreille, 1804	3.78 ± 0.98*	12
Araneae/Lycosidae	*Pardosa* sp.	1.18 ± 0.40**	35
Collembola/Entomobryidae	*Sinella curviseta* Brook, 1882	1.50 ± 0.00*	19
Dictyoptera/Blattellidae	*Symploce pallens* (Stephens, 1835)	2.66 ± 0.37*	20
Isoptera/Rhinotermitidae	*Reticulitermes santonensis* Feytaud, 1924	3.76 ± 0.57*	25
Ensifera/Gryllidae	*Acheta domestica* (Linnaeus 1758)	3.60 ± 0.27*	25
Lepidoptera/Pyralidae	*Ephestia kuehniella* Zeller, 1879; caterpillars	4.56 ± 0.98*	19
Hymenoptera/Formicidae	*Lasius niger* (Linnaeus, 1758)	3.07 ± 0.12*	19
Diptera/Drosophilidae	*Drosophila melanogaster* Meigen, 1830; *Drosophila hydei* Sturtevant, 1921	2.00 ± 0.32*	32
Coleoptera/Chrysomelidae	*Callosobruchus maculatus* (Fabricius, 1775)	3.00 ± 0.27*	21

The differences in acceptance rates for ten prey types were analysed using Generalised Estimating Equations (GEE) with binomial errors from the geepack package^44^. GEE is an extension of the Generalised linear model (GLM) for correlated data. It was used because there were repeated measurements on each individual spider^45^. An autoregressive correlation structure (AR1) for replicated observations over time was used to account for these temporal replications. Subsequently, the relative frequency of the acceptance of each prey type was compared to the average prey acceptance for all prey types using a binomial test. The standardized Levins’ index (*B*_*A*_) of niche breadth^46^ was used to calculate the fundamental trophic niche breadth. Values of *B*_*A*_ higher than 0.6 indicate a wide niche; values below 0.4 indicate a narrow niche^47^.

### Efficiency of prey capture

To measure the capture efficiency for differently sized prey, wolf spiders (*Pardosa* sp.) of various sizes and developmental stages were randomly offered to *Poecilochroa* in a similar manner to the previous acceptance trials. Individuals of *Poecilochroa* were placed singly in Petri dishes and offered the prey item after 1 h of acclimation. If the prey was not accepted within one hour, it was replaced by a smaller one (on average two times smaller than the previous prey item). If a spider did not accept smaller prey, it was considered to be unmotivated to eat and such data were discarded. The length of the prosoma in all spiders was measured under a LEICA EZ5 binocular lens with an ocular micrometer. In total, 84 trials with 37 juvenile individuals of *Poecilochroa* were performed. The logit model with binomial distribution using GEE was used to fit the binary data, as there were repeated measurements on each individual spider. An AR1 correlation structure was used to account for the temporal replications.

In addition, 45 trials using freshly hatched first instar juveniles of both *Poecilochroa* and *Mogrus* were performed. One week before the experiment, each spider was fed with a *Drosophila* fly. In each trial, two individuals, one of each species, were placed in a Petri dish (diameter 35 mm) and observed until one of the spiders was killed by the other. Subsequently, the hunting success of *Poecilochroa* or *Mogrus* was recorded. The size of the prosoma of all juvenile spiders was measured under a LEICA EZ5 binocular lens with an ocular micrometer.

### Nest usurpation

To find how *Poecilochroa* penetrates jumping spider nests, interactions between *Poecilochroa* and *Mogrus* were staged on *Mogrus* nests. Female *Mogrus* and *Poecilochroa* spiders were fed five days before the trials with *Drosophila* flies *ad libitum*. Occupied nests of *Mogrus* on their supporting branches were placed in transparent plastic boxes (55 × 55 × 75 mm) and acclimated for at least 24 hours. Then, a female or juvenile *Poecilochroa* was introduced on the branch above the nest and the interactions between the two spiders were recorded on a camcorder (Canon Leigra HF R56). The recording ended one hour after the introduction of *Poecilochroa*. If *Mogrus* was not captured within 1 hour, the outcome of the interaction was recorded the following day, after an additional 23 hours. In total, 32 observations were made on occupied *Mogrus* nests and the behaviours of *Poecilochroa* and responses of *Mogrus* were described (Table 1).

The effect of the relative sizes of the prey and predator on usurpation success was analysed using GEE. The logit model with binomial distribution and an AR1 correlation structure was used to account for temporal replications, as several *Poecilochroa* individuals were used more than once. The capture efficiency on *Mogrus* was compared with that of wolf spiders as prey.

In addition, 19 observations were conducted of *Poecilochroa* behaviour in response to empty *Mogrus* nests. The nests were placed in transparent plastic boxes (55 × 55 × 75 mm) and the resident *Mogrus* spiders were removed. Then, female or juvenile *Poecilochroa* spiders, fed five days before the trials with a surplus of *Drosophila* flies, were introduced into the boxes with empty nests. The presence of *Poecilochroa* inside or outside the nest was documented after one hour and 24 hours.

### Predatory behaviour

To observe how *Poecilochroa* can overcome spider prey, predatory encounters between *Poecilochroa* and *Mogrus* were staged. The mean prosoma size ratio of *Mogrus* and *Poecilochroa* spiders in this experiment was 1.00 ± 0.14. Spiders were fed five days before trials with *Drosophila* flies *ad libitum*. At the start of each trial, spiders were put individually into small elongated glass tubes (length 35 mm, diameter 6 mm) to simulate the narrow space inside a *Mogrus*’ nest. Then, the two tubes, one hosting *Poecilochroa* and the other hosting *Mogrus*, were connected by their openings and the interaction between the spiders was video-recorded. If the interaction did not result in predatory behaviour within 30 minutes, the trial was ended. Hunting sequences were recorded either on a camcorder (Canon Leigra HF R56, 22 trials) or BW high speed camera (IDT MotionXtra N3, 15 trials) at 50 FPS, to record the details of the attack. In total, 34 complete hunting sequences out of 37 recordings were obtained. In the complete hunting sequences, the different behaviours of *Poecilochroa* were distinguished (Table 2).

The effect of the relative sizes of the prey and predator on the number of “silk swathing” events in individual sequences was analysed using GEE with Poisson distribution and an AR1 correlation structure to account for temporal replications, as several *Poecilochroa* individuals were used more than once. Piriform silk on captured *Mogrus* individuals was visualized by means of fluorescent dye following the protocol by Johnson *et al*.^48^.

## Supplementary information

Supplementary material

Video S1

Video S2
