# Supplementary material for: Nest usurpation: a specialised hunting strategy used to overcome dangerous spider prey
Source: Sci Rep. 2019 Mar 29;9:5386. doi: 10.1038/s41598-019-41664-6 (PMC6441022; doi:10.1038/s41598-019-41664-6)
Supplement: Supplementary file 1 — Supplementary material [file 41598_2019_41664_MOESM1_ESM.docx]

**Nest usurpation: a specialised hunting strategy used to overcome dangerous spider prey**

**Authors:** Ondřej Michálek, Yael Lubin, Stano Pekár

**Supplementary material**

**Video S1.** Usurpation of *Mogrus logunovi* nest by *Poecilochroa senilis* recorded on a camcorder (Canon Leigra HF R56).

**Video S2.** Predatory behaviour of *Poecilochroa senilis* while overcoming *Mogrus logunovi* in a narrow space recorded on a BW high speed camera (IDT MotionXtra N3) at 50 FPS.
